# p21-activated kinase 1 determines stem-like phenotype and sunitinib resistance via NF-*κ*B/IL-6 activation in renal cell carcinoma

**DOI:** 10.1038/cddis.2015.2

**Published:** 2015-02-12

**Authors:** Y Zhu, H Liu, L Xu, H An, W Liu, Y Liu, Z Lin, J Xu

**Affiliations:** 1 Department of Urology, Ninth People's Hospital, School of Medicine, Shanghai Jiaotong University, Shanghai 200011, China; 2 Key Laboratory of Glycoconjugate Research, MOH, Department of Biochemistry and Molecular Biology, School of Baskic Medical Sciences, Shanghai Medical College of Fudan University, Shanghai 200032, China; 3 Department of Urology, Zhongshan Hospital, Fudan University, Shanghai 200032, China

## Abstract

The p21-activated kinase 1 (PAK1), a serine/threonine kinase that orchestrates cytoskeletal remodeling and cell motility, has been shown to function as downstream node for various oncogenic signaling pathways to promote cell proliferation, regulate apoptosis and accelerate mitotic abnormalities, resulting in tumor formation and invasiveness. Although alterations in PAK1 expression and activity have been detected in various human malignancies, its potential biological and clinical significance in renal cell carcinoma (RCC) remains obscure. In this study, we found increased PAK1 and phosphorylated PAK1 levels in tumor tissues according to TNM stage progression. Elevated phosphorylated PAK1 levels associated with progressive features and indicated unfavorable overall survival (OS) as an independent adverse prognosticator for patients with RCC. Moreover, PAK1 kinase activation with constitutive active PAK1 mutant T423E promoted growth, colony formation, migration, invasion and stem-like phenotype of RCC cells, and vice versa, in PAK1 inhibition by PAK1 kinase inactivation with specific PAK1 shRNA, dead kinase PAK1 mutant K299R or allosteric inhibitor IPA3. Stem-like phenotype due to sunitinib administration via increased PAK1 kinase activation could be ameliorated by PAK1 shRNA, PAK1 mutant K299R and IPA3. Furthermore, nuclear factor*-κ*B (NF*-κ*B)/interleukin-6 (IL-6) activation was found to be responsible for PAK1-mediated stem-like phenotype following sunitinib treatment. Both IL-6 neutralizing antibody and IPA3 administration enhanced tumor growth inhibition effect of sunitinib treatment on RCC cells *in vitro* and *in vivo*. Our results unraveled that oncogenic activation of PAK1 defines an important mechanism for maintaining stem-like phenotype and sunitinib resistance through NF-*κ*B/IL-6 activation in RCC, lending PAK1-mediated NF-*κ*B/IL-6 activation considerable appeal as novel pharmacological therapeutic targets against sunitinib resistance.

Arising from the renal tubular epithelial cells, renal cell carcinoma (RCC) accounts for ∼4% of all malignant diseases and 90% of renal malignancies in adults.^1, 2^ Although most RCCs are detected incidentally by the widespread use of abdominal imaging examinations for unrelated symptoms, ∼25–30% of patients are still diagnosed with metastatic disease.^3^ In addition, 20% of patients with localized RCC undergoing radical surgery experience relapse and develop metastatic RCC (mRCC) during follow-up.^4, 5^ Unfortunately, mRCC is refractory chemotherapy and radiotherapy with a 5-year survival rate of ^<^10%.^6^ Immunotherapy with interleukin 2 (IL-2) and/or interferon-*α* (IFN-*α*) is the standard treatment for mRCC and has limited efficacy by substantial number of adverse effects.^7^ Despite the significant improvement in mRCC treatment with antiangiogenesis drugs such as sunitinib and sorafinib, its duration of therapeutic effect is often short.^8^ Clearly, this dire situation mandates better understanding of the molecular mechanism of RCC carcinogenesis so that novel targets could be identified for effective therapies.

The p21-activated kinases (PAKs) are a family of conversed nonreceptor serine/threonine kinases that function as key regulators of pleiotropic physiological processes including cytoskeleton dynamics and cell polarity, motility, invasion and survival.^9^ Currently, 6 PAKs have been classified into group I PAKs (PAK1–3) and group II PAKs (PAK4–6) on the basis of structural and functional similarities.^10^ As the best-characterized member of the PAK family, PAK1 was identified as a protein that interacts with cell division cycle 42 (CDC42) and RAC1.^11^ In addition to CDC42 and RAC1, other signaling including PI3K/Akt can also lead to the activation of PAK1.^12^ PAK1 phosphorylation at threonine-423 (T423) by upstream signaling has been linked to its activation, as substitution of the acidic residue glutamic acid (E) at this site yields a constitutively active PAK1 T423E enzyme.^13^ Activation and localization of PAK1 lead to mediated physiological effects of downstream signaling via activating additional kinases and other effectors by phosphorylating them at specific serine and threonine residues or through protein–protein interaction.^9^

PAK1 expression and activity are upregulated in different human tumors, such as breast, lung, colorectal, liver and kidney cancers,^14, 15, 16^ and are associated with tumor invasiveness, metastasis and poor prognosis. Besides, PAK1 is also a component of various signaling pathways, including mitogen-activated protein kinase (MAPK), JUN N-terminal Kinase (JNK) and nuclear factor-*κ*B (NF-*κ*B) pathways, all of which are believed to be important in carcinogenesis.^9^ Moreover, PAK1 has been found to play critical roles in anoikis resistance that facilitates metastasis by allowing tumor cells to survive following detachment from the matrix in original tissue and travelling to distant sites. Resistance to anoikis program represents a molecular basis for cancer progression and drug resistance.^15, 17^ The regulation of phosphorylation and function of Snail by PAK1 signaling kinase may contribute to the process of epithelial–mesenchymal transition (EMT) that plays a pivotal role in the conversion of early-stage tumors into invasive malignancies.^18^ EMT induction in cancer cells results in the acquisition of stem-like phenotype and drug resistance trait.^19, 20^

Based on these previous findings, we hypothesized that PAK1-mediated stem-like phenotype might induce sunitinib resistance and involve in RCC tumor progression. Our present study revealed that upregulation of PAK1 kinase activity conferred stem-like phenotype via NF-*κ*B/IL-6 activation *in vitro* and *in vivo* that defines a novel potential mechanism underlying tumor metastasis and sunitinib resistance in RCC patients.

## Results

### High p-PAK1 expression is associated with poor prognosis in patients with RCC

**Figure 1:**
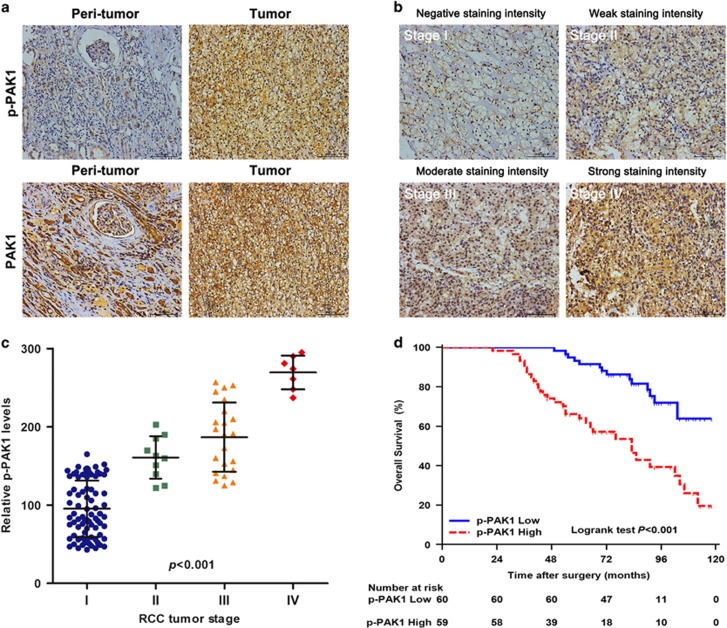
Expression of PAK1 and p-PAK1 in sections of RCC patients. (**a**) Representative microphotographs with PAK1 and p-PAK1 expression in surrounding nontumor tissues (left) and tumor tissues (right) of 119 RCC specimens. Scale bars, 100 *μ*m. (**b**) Representative microphotographs with p-PAK1 gradual ascension during RCC progression from TNM stages I to IV. Scale bars, 100 *μ*m. (**c**) Quantitation of p-PAK1 expression in RCC from TNM stages I to IV. (**d**) Kaplan–Meier survival analysis for OS between RCC patients with high p-PAK1 expression (*n*=59) and low p-PAK1 expression (*n*=60) from a cohort of 119 specimens

O'Sullivan *et al.*^16^ reported that both PAK1 expression and activation correlate with RCC tumor grade *in vivo*. We confirmed these findings by evaluating PAK1 expression and kinase activity in paired tumor and peritumoral specimens from 119 RCC patients. The immunohistochemistry (IHC) staining results showed that expression levels of PAK1 and p-PAK1 were increased in tumor tissues compared with peritumoral tissues (Figure 1a). Moreover, advanced tumors tended to harbor higher p-PAK1 levels (Figures 1b and c), highly consistent with the finding of O'Sullivan *et al.*^16^ In an effort to determine the potential clinical significance of p-PAK1 expression in RCC patients, we quantify the expression levels of p-PAK1 and provide a comparison of clinicopathologic features in dichotomized p-PAK1 status. As summarized in Table 1, p-PAK1 expression was positively correlated with tumor size (*P*<0.001), TNM stage (*P*<0.001), tumor thrombus (*P*=0.013), Fuhrman grade (*P*=0.002) and necrosis (*P*=0.001). To further investigate the prognostic value of p-PAK1 expression for clinical outcome in RCC patients, we applied Kaplan–Meier survival analysis to compare the overall survival (OS) according to p-PAK1 expression. Kaplan–Meier survival analysis indicated that high p-PAK1 expression was associated with reduced OS (*P*<0.001; Figure 1d). Table 2 lists the univariate and multivariate analysis of potential prognostic factors for OS. Univariate analysis revealed that ECOG PS (*P*=0.040), tumor size (*P*=0.003), TNM stage (*P*<0.001), tumor thrombus (*P*<0.001), Fuhrman grade (*P*<0.001) and p-PAK1 expression (*P*<0.001) all significantly influenced OS. Further adjustment of covariate factors by using multivariate Cox analysis identified together with ECOG PS, tumor size, TNM stage, tumor thrombus, and Fuhrman grade and p-PAK1 expression was an independent prognostic factor (*P*=0.029) in RCC patients. These data indicate that p-PAK1 represents a novel biomarker for RCC progression and prognosis.

### PAK1 kinase activity regulates tumorigenic prosperities and maintains stem-like phenotype in RCC cells

**Figure 2:**
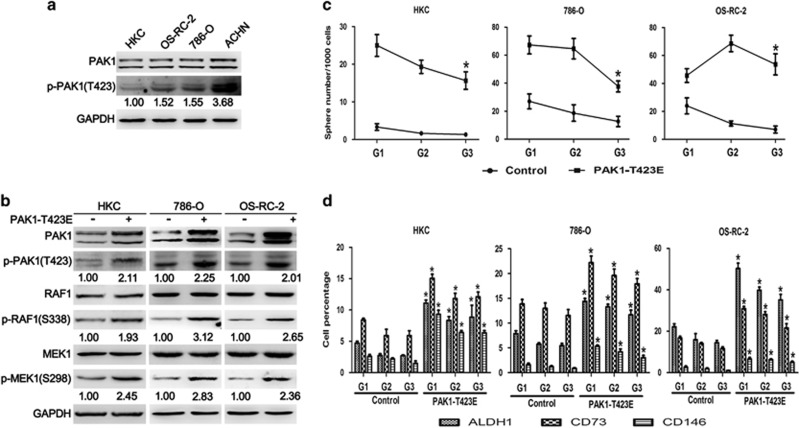
Elevation of PAK1 kinase activity displays stem-like characteristics in RCC cells. (**a**) Western blot analysis of PAK1, p-PAK1 (T423) and GAPDH for HKC, OS-RC-2, 786-O and ACHN cells. GAPDH was used as a loading control. The numbers shown are the ratios of the intensities of the bands for p-PAK1, divided by the intensities of the bands for PAK1, normalized to 1.00 for HKC. (**b**) Western blot analysis of PAK1 and p-PAK1 (T423), RAF1, p-RAF1(S338), MEK1, p-MEK1(S298) and GAPDH for HKC, 786-O and OS-RC-2 cells stably transfected with PAK1 T423E. GAPDH was used as a loading control. The numbers shown are the ratios of the intensities of the bands for pPAK1(T423), p-RAF1(S338) and p-MEK1(S298), divided by the intensities of the bands for GAPDH, RAF1 and MEK1, respectively, normalized to 1.00 for cell lines without PAK1 T423E transfected. (**c**) Analysis of self-renewal of HKC, 786-O and OS-RC-2 cells stably transfected with control (empty vector) or PAK1-T423E. Data are represented as means±S.D. of triplicate experiments. *P<0.05 *versus* control. (**d**) Flow cytometry analysis of ALDH1, CD73 and CD146 for HKC, 786-O and OS-RC-2 cells stably transfected with control or PAK1 T423E from generations G1 to G3 spheres. Data are represented as means±S.D. of triplicate experiments. *P<0.05 *versus* control

O'Sullivan *et al.*^16^ demonstrated that increased PAK1 and PAK1 kinase activity played a significant role in tumorigenic phenotype in human RCC both *in vitro* and *in vivo*. As shown in Figure 2a, different RCC cell lines including OS-RC-2, 786-O and ACHN exhibited higher protein levels of p-PAK1 compared with normal renal proximal tubular epithelial cell line (HKC). We further evaluated the functional effect of PAK1 kinase activity on RCC cells. 786-O and OS-RC-2 were stably transfected with constitutively active mutant PAK1 T423E expression plasmid, respectively (Figure 2b). The pathway downstream PAK1 was activated that confirmed with phosphorylation of specific PAK1-dependent residues on MEK1 (see Frost *et al.*^22^ and Beeser *et al.*^22^) and RAF1 (see Jin *et al.*^23^) by western blot (Figure 2b). PAK1 kinase activity promotes 786-O and OS-RC-2 cell proliferation, anchorage-independent growth, migration and invasion ability (Supplementary Figure 1). In order to demonstrate the dependency of the observed phenotypes on PAK1 activity, HKC cells were stably transfected with PAK1 T423E (Figure 2b). We confirmed that the same observed phenotypes on PAK1 activity were reproduced in HKC cells (Supplementary Figure 1). The assessment of self-renewal in cell lines showed that active PAK1 T423E stably transfected cells not only gained the ability to initiate sphere growth in serum-free conditions but maintained it for at least three generations (Figure 2c). However, the empty vector transfected cells possessed only a limited lifespan in 3D culture. The extent of sphere formation is also considered to be correlated with the stem-like cell content of tumor cell populations.^24^ Acquisition of long-term self-renewal in PAK1 T423E stably transfected cells correlated with a stable upregulation of cancer stem cell (CSC) markers including CD73, CD146,^25^ and aldehyde dehydrogenase 1 (ALDH1).^26^ Conversely, the empty vector transfected cells were losing the CSC markers expression along with sphere passaging (Figure 2d and Supplementary Figure 2). Collectively, these findings suggest that elevated PAK1 kinase activity contributes to the stem-like phenotype in RCC cells.

### Tumorigenicity and stem-like phenotype are suppressed by the inhibition of PAK1 kinase activity in RCC cells

**Figure 3:**
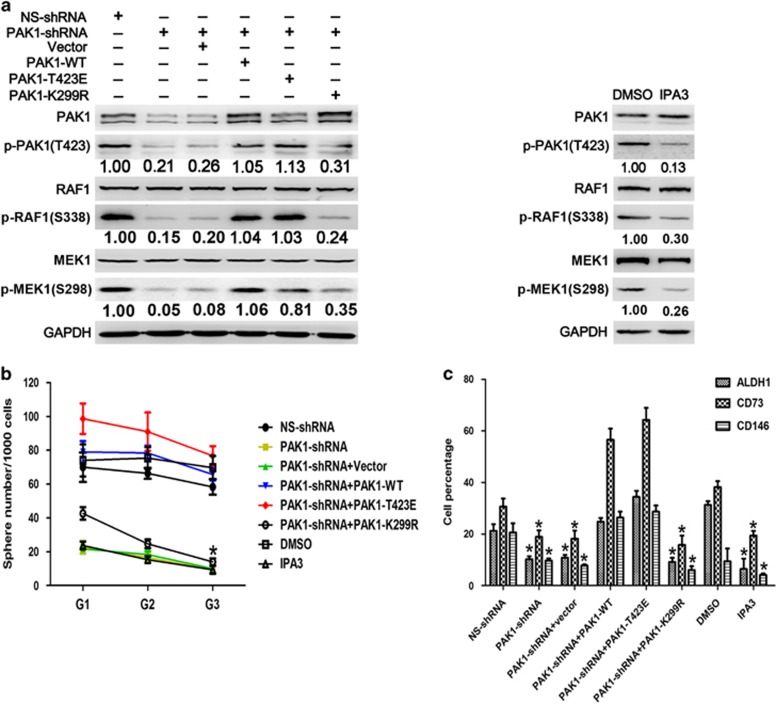
PAK1 kinase activity is necessary for stem-like phenotype in RCC cells. (**a**) Western blot analysis of PAK1, p-PAK1(T423), RAF1, p-RAF1(S338), MEK1, p-MEK1(S298) and GAPDH for ACHN cells stably transfected with NS-shRNA or PAK1-shRNA stably transfected with vector (empty vector), PAK1-WT, PAK1-T423E, and PAK1 K299R (left panel) and treated without or with IPA3 (7.5 *μ*M; right panel). GAPDH was used as a loading control. The numbers shown are the ratios of the intensities of the bands for p-PAK1(T423), p-RAF1(S338) and p-MEK1(S298), divided by the intensities of the bands for GAPDH, RAF1 and MEK1, respectively, normalized to 1.00 for ACHN cells stably transfected with NS-shRNA or treated with DMSO. (**b**) Analysis of self-renewal of ACHN cells with above-mentioned treatment. Data are represented as means±S.D. of triplicate experiments. *P<0.05 *versus* NS-shRNA and DMSO, respectively. (**c**) Flow cytometry analysis of ALDH1, CD73 and CD146 for ACHN cells with above-mentioned treatment from generations G3 spheres. Data are represented as means±S.D. of triplicate experiments. *P<0.05 *versus* NS-shRNA and DMSO, respectively

It has been reported that signaling through PAK1 triggered by interaction with small GTPases induces cell migration, invasion, survival as well as a number of other cellular processes.^9, 27^ Thus, we analyze the pivotal role of PAK1 kinase activity in RCC progression. We found that shRNA depletion of PAK1 induced a significant decrease in phosphorylation levels of RAF1 (S338) and MEK1 (S298) compared with control ACHN cells. This defect in PAK1 activity was rescued by either a shRNA-resistant wild-type PAK1 or a PAK1 T423E mutant, but not rescued by a shRNA-resistant PAK1 K299R mutant. In addition, PAK1 kinase activity was inhibited by allosteric inhibitor IPA3 treatment in ACHN cell line (Figure 3a). Next, shRNA depletion of PAK1 led to a striking decrease in the anchorage-dependent and -independent growth, migration and invasion ability in ACHN cells that was rescued by either a shRNA-resistant wild-type PAK1 or a PAK1 T423E mutant, but not by a shRNA-resistant PAK1 K299R mutant. Similar phenomenon was also found by IPA3 treatment (Supplementary Figure 3). Depletion of PAK1 prohibited acquisition of self-renewal in ACHN cells (Figure 3b), and significantly decreased the expression of CSC markers (Figure 3c and Supplementary Figure 4A). Self-renewal and CSC marker expression were rescued by either shRNA-resistant PAK1 or PAK1 T423E, but not shRNA-resistant PAK1 K299R. In addition, IPA3 treatment induced the same phenomenon (Figures 3b and c and Supplementary Figure 4B). Consistently, these experiments implicate that PAK1 kinase activity plays a role in tumor progression and stem-like phenotype in RCC.

### Sunitinib-induced PAK1 activation leads to stem-like phenotype and sunitinib resistance *in vitro*

**Figure 4:**
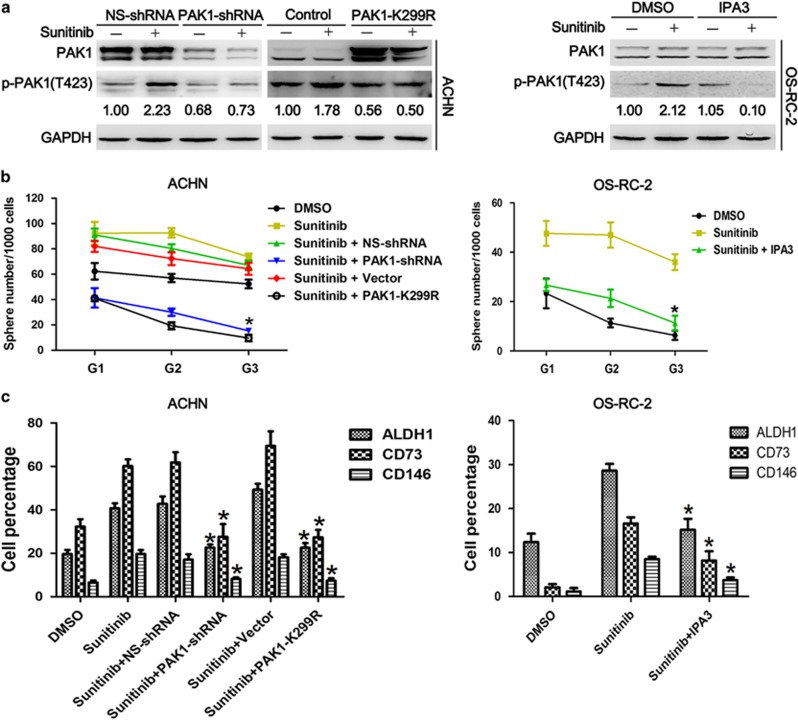

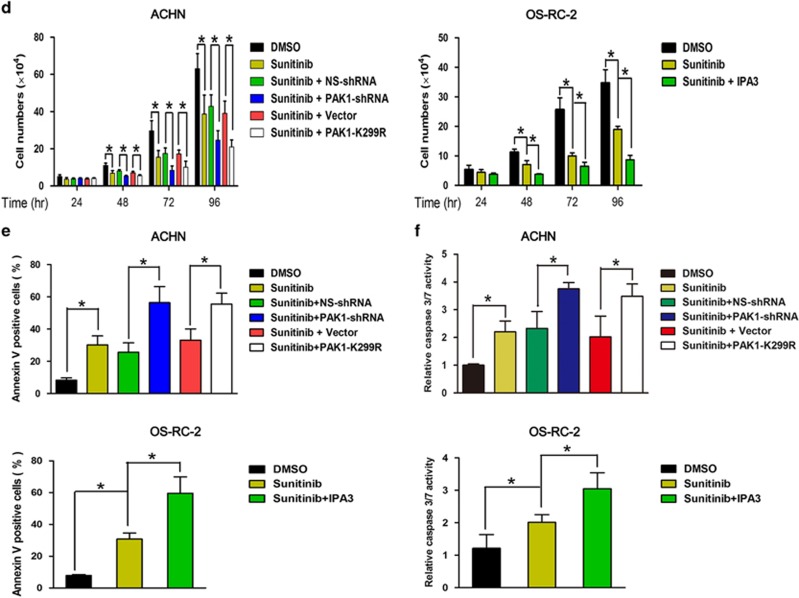
PAK1 kinase activity inhibition enhances sunitinib sensitivity in RCC cells. (**a**) Western blot analysis of PAK1, p-PAK1(T423) and GAPDH for ACHN cells stably transfected with NS-shRNA and PAK1-shRNA-1 without or with sunitinib (5 *μ*M) treatment for 48 h (left panel), for ACHN cells stably transfected with control (empty vector) or PAK1-K299R without or with sunitinib (5 *μ*M) treatment for 48 h (middle panel), for OS-RC-2 cells in the presence with IPA3 (7.5* μ*M) with or without sunitinib (5 *μ*M) treatment for 48 h (right panel). GAPDH was used as a loading control. The numbers shown are the ratios of the intensities of the bands for p-PAK1(T423) divided by the intensities of the bands for GAPDH, normalized to 1.00 for ACHN cells transfected with NS-shRNA, and OS-RC-2 cells without treatment with sunitinib, respectively. (**b**) Analysis of self-renewal of ACHN and OS-RC-2 cells with above-mentioned treatment. Data are represented as means±S.D. of triplicate experiments. *P<0.05 *versus* sunitinib. (**c**) Flow cytometry analysis of ALDH1, CD73 and CD146 for ACHN and OS-RC-2 cells with above-mentioned treatment from generations G3 spheres. Data are represented as means±S.D. of triplicate experiments. *P<0.05 *versus* sunitinib. (**d–f**) Cell proliferation assay (**d**), annexin V/PI staining assay (**e**) and caspase 3/7 activity (**f**) for ACHN and OS-RC-2 cells with above-mentioned treatment. Data are represented as means±S.D. of triplicate experiments. *P<0.05

Consistent with previous study that indicated that cancer stem-like cells are usually resistant to conventional chemotherapy and radiotherapy compared with differentiated cancer cells,^28^ our aforementioned data showed that PAK1 kinase activity increased stem-like phenotype in RCC cells, and thus we hypothesize that sunitinib resistance is due, at least in part, to the accumulation of stem-like phenotype after sunitinib administration. Western blot analysis showed that sunitinib treatment upregulated p-PAK1 expression that could be reversed by PAK1 knockdown, PAK1 K299R plasmid transfection in ACHN cells or IPA3 co-treatment in OS-RC-2 cells (Figure 4a). In addition, self-renewal and CSC marker expression were significantly increased in the treatment of sunitinib that were ablated by either PAK1 knockdown or PAK1 K299R transfected in ACHN cells or IPA3 treatment in OS-RC-2 cells (Figures 4b and c and Supplementary Figure 5). Therefore, we wondered whether inhibition of PAK1 activity could enhance sunitinib sensitivity in RCC cells. Cell proliferation assay showed that RCC cells with inhibited PAK1 kinase activity exhibited lower growth rate in the presence of sunitinib compared with sunitinib alone (Figure 4d). Ong *et al.*^29^ provided evidence for targeting PAK1 to induce apoptosis of tumor cells in breast cancer and squamous NSCLCs. Annexin V staining and caspase 3/7 activity assay were then performed to determine whether the decrease in proliferation was due to cell apoptosis. Our results show that, in the presence of sunitinib, cells with inhibited PAK1 kinase activity displayed most apoptotic cells (Figures 4e and f). Taken together, these data show that p-PAK1 could promote stem-like phenotype and therefore reduce the efficacy of sunitinib *in vitro*. However, the underlying mechanisms need to be further elucidated.

### NF-*κ*B/IL-6 signaling functions downstream of PAK1 in the regulation of stem-like phenotype and sunitinib resistance

**Figure 5:**
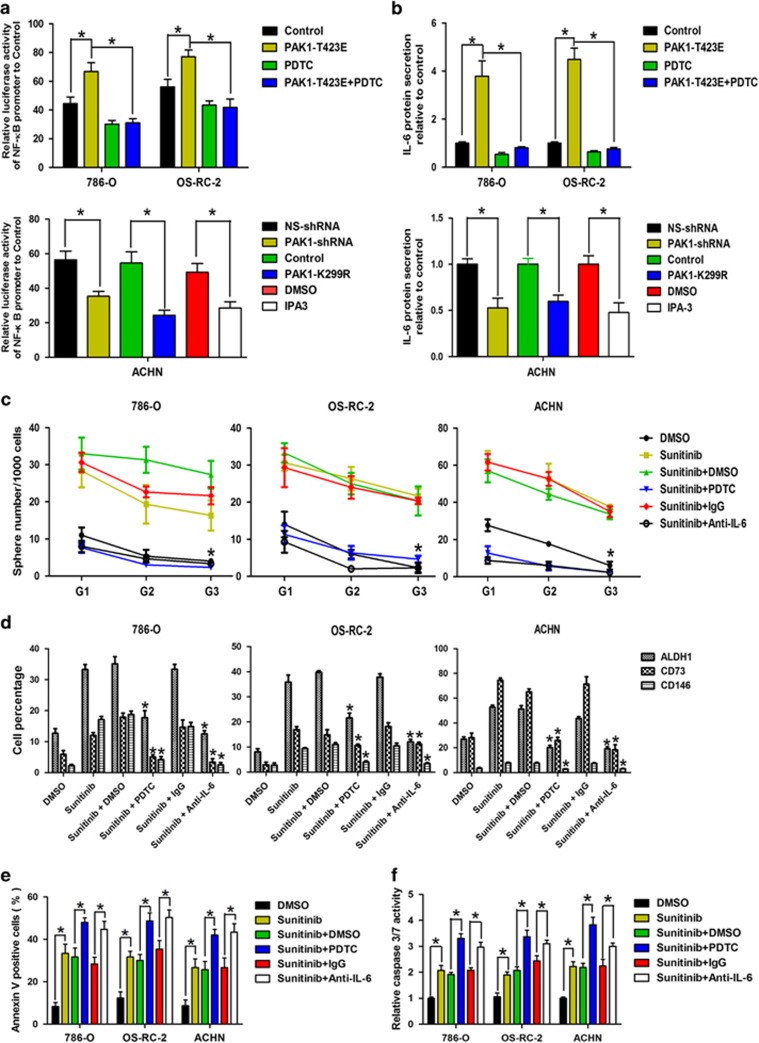
PAK1-mediated stem-like phenotype and sunitinib resistance depend on NF-*κ*B/IL-6 activation. (**a**) Luciferase activity assay for NF*-κ*B promoter transcription analysis in 786-O and OS-RC-2 cells after PAK1 T423E transfection pretreatment without or with PDTC (10 *μ*M) (upper), in ACHN cells stably transfected with NS-shRNA, PAK1-shRNA, control (empty vector) and PAK1-K299R, respectively, or treatment without or with IPA3 (7.5 *μ*M) (lower). Data are represented as means±S.D. of triplicate experiments. *P<0.05. (**b**) The secretion of IL-6 in 786-O, OS-RC-2 and ACHN with above-mentioned treatment. Data are represented as means±S.D. of triplicate experiments. *P<0.05. (**c**) Analysis of self-renewal of 786-O, OS-RC-2 and ACHN cells with above-mentioned treatment. Data are represented as means±S.D. of triplicate experiments. *P<0.05 *versus* sunitinib. (**d**) Flow cytometry analysis of ALDH1, CD73 and CD146 for 786-O, OS-RC-2 and ACHN cells with above-mentioned treatment from generations G3 spheres. Data are represented as means±S.D. of triplicate experiments. *P<0.05 *versus* sunitinib. (**e**) Annexin V/PI staining assay and (**f**) caspase 3/7 activity for 786-O, OS-RC-2 and ACHN cells with above-mentioned treatment. Data are represented as means±S.D. of triplicate experiments. *P<0.05

Activated PAK1 causes NF-*κ*B activation and secretion of proinflammatory cytokines in epithelial cells.^30^ Furthermore, NF-*κ*B mediates tumor progression and resistance to anticancer therapeutics by regulating stem-like activities.^31^ We speculated that NF-*κ*B signaling might be involved in PAK1-mediated stem-like phenotype in RCC. Luciferase activity assay showed that enhanced PAK1 kinase activity by PAK1 T423E plasmid transfection increased NF-*κ*B promoter transcription activity that could be reserved by NF-*κ*B-specific inhibitor (PDTC) in 786-O and OS-RC-2 cells (Figure 5a, upper panel). Conversely, PAK1 knockdown, PAK1 K299R plasmid transfection and IPA3 treatment in ACHN cells significantly decreased NF-*κ*B promoter transcription activity (Figure 5a, lower panel). NF-*κ*B synergistically promotes the transcription of inflammatory cytokines such as IL-6 and IL-8.^32^ ELISA assay showed that IL-6 secretion was increased after PAK1 T423E plasmid transfection that could be reserved by PDTC in 786-O and OS-RC-2 cells (Figure 5b, upper panel). In addition, IL-6 secretion was decreased following PAK1 inhibition in ACHN cells (Figure 5b, lower panel). Furthermore, Krishnamurthy *et al.*^33^ reported that endothelial cell IL-6 enhanced orosphere formation, p-STAT3 activation, survival and self-renewal of human CSC. Liu *et al.*^34^ showed that IL-6 enriched lung cancer stem-like cell population. To further investigate the role of NF-*κ*B/IL-6 signaling in PAK1-mediated stem-like phenotype, 786-O, OS-RC-2 and ACHN cells were treated with PDTC or IL-6 neutralizing antibody (anti-IL-6) in the presence of sunitinib. Either PDTC or IL-6 neutralizing antibody treatment could repress sunitinib-induced tumor cell self-renewal and CSC marker expression (Figures 5c and d and Supplementary Figure 6). In addition, annexin V staining and caspase 3/7 activity assay showed that RCC cells with NF-*κ*B/IL-6 inhibited were more sensitive to sunitinib treatment (Figures 5e and f). These results demonstrate that sunitinib-induced stem-like phenotype and sunitinib resistance could be reversed, at least in part, by inhibiting PAK1/NF-*κ*B/IL-6 signaling in RCC cells.

### PAK1/NF-*κ*B/IL-6 signaling inhibition leads to increased efficacy of sunitinib *in vivo*

**Figure 6:**
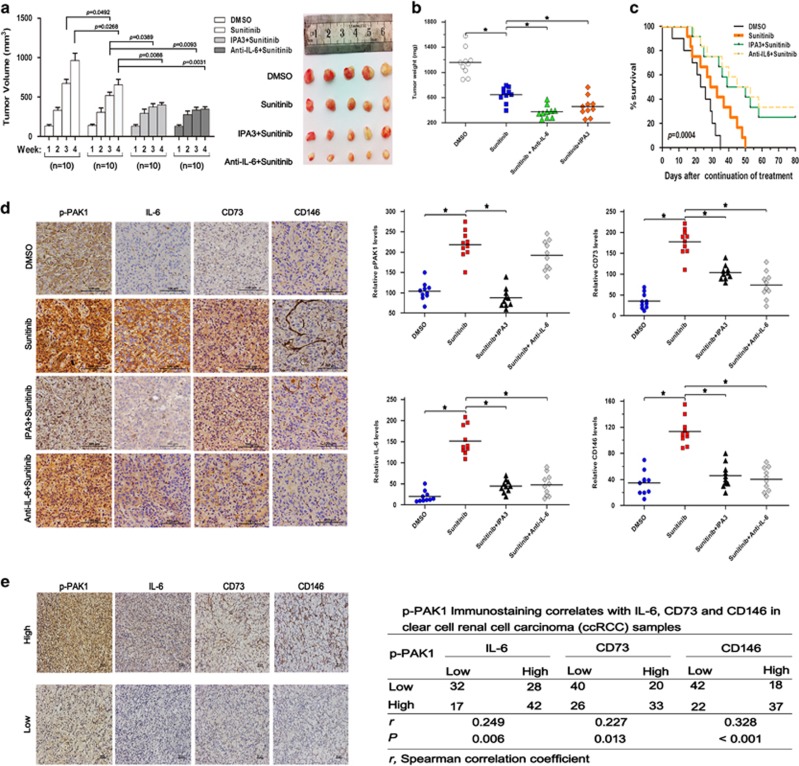
PAK1/NF-*κ*B/IL-6 signaling involved in sunitinib-induced stem-like phenotype *in vivo.* (**a–c**) Subcutaneous tumor images and *in vivo* subcutaneous tumor growth curves (**a**) and total tumor weight (**b**) and overall survival analysis (**c**) (*P=0.0004) of 4 groups of nude mice xenografted with ACHN cells after sunitinib with or without IPA3 (7.5 *μ*M) or anti-IL-6 treatment for 4 weeks are shown. (**d**) Representative IHC staining images for p-PAK1, IL-6, CD73 and CD146 expression in xenograft RCC tumor tissues from above-mentioned groups (left). Quantified relative p-PAK1, IL-6, CD73 and CD146 expression levels in the above-mentioned groups (right). (**e**) Representative microphotographs with p-PAK1, IL-6, CD73 and CD146 expression in RCC tumor tissues of 119 RCC specimens. Scale bars, 50 *μ*m (left). Correlation of expression levels of p-PAK1, IL-6, CD73 and CD146 in RCCs (right)

We sought to determine whether inhibiting PAK1/NF*-κ*B/IL-6 signaling could also lead to increased effectiveness of sunitinib treatment *in vivo*. ACHN cells (with relative higher intrinsic p-PAK1 levels) were injected subcutaneously in nude mice. A week later, the tumor-bearing mice were treated with sunitinib, sunitinib in combination with IPA3 or sunitinib in combination with IL-6 neutralizing antibody. As expected, sunitinib treatment inhibited tumor formation of ACHN cells in nude mice. More importantly, the efficacy of sunitinib was enhanced significantly when combined with IPA3 or IL-6 neutralizing antibody (Figures 6a–c). Interestingly, representative IHC images and quantification of IHC data of tumor tissues from the nude mice showed that sunitinib treatment increased p-PAK1, IL-6, CD73 and CD146 staining (Figure 6d). Consistent with our *in vitro* data, sunitinib in combination with IPA3 or IL-6 neutralizing antibody could significantly decrease the expression of RCC stem cell markers (Figure 6d). Importantly, p-PAK1 immunostaining in the human RCC specimens showed significant positive correlations with IL-6 (*P*=0.006), CD73 (*P*=0.013) and CD 146 staining (*P*<0.001) (Figure 6e). Collectively, these data indicate that sunitinib-induced stem-like phenotype *in vivo* could be attributed, at least in part, to PAK1/NF-*κ*B/IL-6 activation and that simultaneous PAK1/NF-*κ*B/IL-6 inhibition could synergize sunitinb treatment.

## Discussion

Currently, no approved therapies could get duration of therapeutic effect in clinical RCC treatment. Determining the potential mechanisms of RCC carcinogenesis may identify new therapeutic approaches. Herein, we used 119 RCC specimens to illustrate the association between p-PAK1 expression and overall survival after nephrectomy. The present study demonstrated that intratumoral p-PAK1 overexpression significantly correlated with more aggressive tumor behavior and poor prognosis. To our knowledge, this is the first study to indicate p-PAK1 as an independent prognosticator for overall survival in clinical RCC patients. Furthermore, our work has established for the first time that sunitinib-induced upregulation of PAK1 kinase activity and ensuing NF-*κ*B/IL-6 activation contributed to the accumulation of stem-like cancer cells, RCC progression and sunitinib resistance. These findings warrant further evaluation of p-PAK1 as a predictive biomarker for sunitinib response and resistance in RCC treatment and establish the evolving paradigm of using PAK1-targeted agents to assist antiangiogenic therapies.

O'Sullivan *et al.*^16^ and our group found that PAK1 was upregulated and hyperactivated in RCC, playing an important role in cell proliferation, motility, anchorage-independent growth and drug resistance. Besides, our results herein revealed that intratumoral p-PAK1 overexpression was predictive of poor clinical outcome of RCC patients following surgery, further suggesting the crucial role of PAK1 hyperactivation in RCC progression. Specific upstream signaling including Rho GTPase family members CDC42 and RAC1 dictate the degree of activation and subcellular localization of PAK1, and in turn PAK1 initiates downstream cascades of pathways that culminate in cellular response.^10, 35^ Receptor tyrosine kinases, nonreceptor tyrosine kinases and upstream serine/threonine kinases such as PI3K can also activate PAK1.^9, 12^ Regulating cytoskeletal dynamics, cell adhesion and cell migration by CDC42/RAC1-PAK1 have been reported to play a central role in cancer initiation and progression including the acquisition of unlimited proliferation potential, survival and evasion from apoptosis, tissue invasion and the establishment of metastases.^27, 36, 37^ The upstream signaling of PAK1 in RCC has not been investigated here, and will be addressed in detail in our planned future studies. Activated PAK1 in turn stimulates MAPK pathway in addition to NF-*κ*B pathway to bring about gene expression.^38, 39^ Our current investigation revealed that enhanced PAK1 kinase activity could significantly increase NF-*κ*B activity, whereas PAK1 kinase activity inhibition could obviously decrease NF-*κ*B activity in RCC cells. This effect is consistent with a previous study that RCC cells expressing aberrant NF-*κ*B signaling could substantially promote tumor progression and cause resistance to anticancer drugs.^40^

Our previous studies have revealed that anoikis resistance could be conferred by HBX/PAK1, Mgat5/EGFR/PAK1 and Klotho/VEGFR2/PAK1 activation in hepatoma cells.^41, 42, 43^ Moreover, Chen *et al.*^44^ have demonstrated that C-terminal kinase domain of the p34cdc2-related PITSLRE protein kinase (p110c) associated with PAK1 and inhibits its activity during anoikis; Menard *et al.*^17^ and Guo *et al.*^45^ have showed that active PAK1 could rescue cells from undergoing anoikis. Our current investigation revealed that upregulation of PAK1 kinase activity increased RCC cell anchorage-independent growth, reflecting enhanced ability to resist anoikis. PAK1 was also suggested to promote the progression of EMT by directly phosphorylating Snail in breast cancer cells.^18^ Lv *et al.*^46^ reported that RAC1/PAK1 signaling promotes EMT of podocytes *in vitro* via triggering *β*-catenin transcriptional activity under high glucose conditions. Mani *et al.*^20^ have found that use of genetic or pharmacologic techniques to transiently induce an EMT in large populations of differentiated normal epithelial cells may generate cells with properties of stem cells. The molecular events linking intrinsic (e.g., levels of transcription factors, signaling pathways) with extrinsic influences (e.g., host factors, microenvironment, immune response) may play an important role in rendering stem-like phenotype of cancer cells with the ability to modulate tumorigenicity and drug responses.^47, 48^ Our present study suggested that PAK1 signaling had an impact on cancer cell behavior and stem-like phenotype. It has been reported that NF-*κ*B could mediate the secretion of proinflammatory cytokines (IL-6 and IL-8) in various types of cancer.^49^ More recently, a study showed that IL-6 could induce highly oncogenic, drug-resistant, stem-like phenotype in cancer cells.^50^ Our data herein elucidated a novel PAK1/NF-*κ*B/IL-6 signaling pathway that might be partly responsible for the transformation of a stem-like phenotype in RCC cells.

Resistance and low responsiveness to antiangiogenic tyrosine kinase inhibitors such as sunitinib is a major clinical obstacle in the treatment of mRCC.^51^ Therefore, uncovering the underlying mechanisms has profound clinical significance. Huang *et al.*^52^ observed that IL-8 is an important contributor to sunitinib resistance in RCC. Hammers *et al.*^53^ suggested that reversible EMT may be associated with acquired tumor resistance to sunitinib in patients with RCC. Shojaei *et al.*^54^ showed that HGF/c-Met pathway also played a role in the development of resistance to antiangiogenic therapy. Gotink *et al.*^55^ found lysosomal sequestration to be a novel mechanism of sunitinib resistance. More recently, Bender and Ullrich^56^ proposed that PRKX, TTBK2 and RSK4 expression causes sunitinib resistance in RCC. Consistent with the excellent work of Chinchar *et al.*^57^ that sunitinib significantly suppresses proliferation but increases breast cancer stem cells, we found that sunitinib-induced upregulation of PAK1 kinase activity enhanced the RCC stem-like phenotype. These findings suggested that PAK1 activation improved stem cell properties rather than increased proliferation rates, leading to higher number of cancer cell spheres. Moreover, Soeda *et al.*^24^ reported that intratumoral hypoxia promoted the self-renewal capacity of CD133-positive human glioma-derived cancer stem cells. Pàez-Ribes *et al.*^58^ showed that anti-angiogenic drugs increased local invasion and distant metastasis due to antiangiogenesis-induced intratumoral hypoxia by blocking neovascularization. Thus, we hypothesized that the enhanced RCC stem-like phenotype by sunitinib was possibly because of antiangiogenesis-induced intratumoral hypoxia. The relationship between sunitinib, antiangiogenesis-induced intratumoral hypoxia, PAK1 kinase activity and RCC stem-like phenotype would be further investigated in our future study. Here, our findings identified that sunitinib-induced upregulation of PAK1 kinase activity and downstream NF-*κ*B/IL-6 activation could be an important mechanism of sunitinb resistance.

In conclusion, our results indicate that upregulation of PAK1 kinase activity and its downstream signaling contributes to the stem-like phenotype, tumor progression and sunitinib resistance in RCC. Inhibiting PAK1/NF-*κ*B/IL-6 activation might be a promising strategy to reverse sunitinb resistance or enhance the efficacy of antiangiogenic agents for RCC patients.

## Materials and Methods

### Cell lines and human samples

Three human RCC cell lines, 786-O, OS-RC-2 and ACHN, were obtained from Shanghai Cell Bank (Shanghai, China). One human renal proximal tubular epithelial cell line HKC was provided by Dr. Donghai Wen (Fudan University, Shanghai, China). Cells were cultured in DMEM or RPMI 1640 supplemented with 10% FBS at 37°C in a humidified 5% CO_2_ incubator. These cell lines have been characterized at the bank by DNA fingerprinting analysis using short tandem repeat markers. Tumor and peritumoral samples obtained from 119 patients with RCC undergoing nephrectomy are described in detail in the Supplementary Information.

### Plasmid transfection and RNA interference

The plasmids containing NF-*κ*B luciferase reporter (pGL3-NF-*κ*B) were generated as previously described.^59^ Expression plasmids encoding wild-type PAK1, constitutively-active PAK1 (T423E) and dominant-negative PAK1 (K299R) were constructed as previously described.^15^ All plasmid constructs were confirmed by DNA sequencing. Transient and stable transfections with various plasmids and corresponding empty vectors as control were performed as before and described in the Supplementary Information.^41^ The shRNA to PAK1 (5′-CGAGGAACCTGGTCTCCGCATCCAGTTAC-3′) was obtained from Origene Technologies (Rockville, MD, USA). Gene silencing effect was confirmed by western blot analysis and RT-PCR at 72 h after transfection. To rescue PAK1 expression we modified the PAK1 cDNA to be resistant to shRNA by changing the targeted site to 5′-*AGA*GG*T*AC*A*TGG*AGC*CC*T*CATCCAGTTAC-3′, with synonymous changes italicized.

### Western blot analysis and ELISA assay

Western blot and ELISA were carried out as previously described.^60^ Primary antibodies used in western blot included those against PAK1, p-PAK1(T423), GAPDH (Santa Cruz Biotechnology, Santa Cruz, CA, USA) MEK1, p-MEK1(S298) and RAF1, p-RAF1 (S338) (Cell Signaling Technology, Beverly, MA, USA). ELISA was performed with Human IL-6 ELISA kit (R&D Systems, Minneapolis, MN, USA) according to the manufacturer's instructions.

### Tumorsphere assay

Single cell was seeded on ultra-low attachment plates (Corning, Corning, NY, USA) at a concentration of 3000 cells/ml in DMEM/F12 medium (Invitrogen, Carlsbad, CA, USA) supplemented with B27 (1 : 50, GIBCO, Grand Island, NY, USA), 20 ng/ml EGF (PeproTech, Rocky Hill, NJ, USA) and 10 ng/ml basic FGF (PeproTech). Tumorspheres >50 *μ*m in diameter were counted 7 days after seeding. The first generations of 10-day-old spheres were collected by gravity and dissociated to single-cell suspension with trypsin for serial passaging (G1–G3).

### Proliferation and viability

Cells were plated at a density of 3 × 10^4^ cells/well into a 6-well tissue culture plate. Cell proliferation was performed by Z2 counter and viability by Vi Cell XR 2.03 (Beckman Coulter, Fullerton, CA, USA) using Trypan Blue exclusion staining at 24, 48, 72 and 96 h.

### ALDEFLUOR assay

ALDH activity was detected using the ALDEFLUOR assay kit (Stem Cell Technologies, Vancouver, BC, Canada) as described by the manufacturer. Briefly, dissociated single cells from cell lines were suspended in ALDEFLUOR assay buffer containing an ALDH substrate, bodipy-aminoacetaldehyde (BAAA), at 1.5 *μ*M and incubated for 1 h at 37°C. A specific inhibitor of ALDH, diethylaminobenzaldehyde (DEAB), at a 10-fold molar excess, was used as negative control. Flow cytometry data were analyzed by FlowJo software (TreeStar, Ashland, OR, USA).

### Colony formation assay, cell migration assay, cell invasion assay, flow cytometry analysis, annexin V staining and caspase 3/7 activity assay

Colony formation assay, cell migration assay, cell invasion assay and flow cytometry analysis, annexin V staining and caspase 3/7 activity assay were performed as before or according to the manufacturer's instructions and described detailed in the Supplementary Information.^60^

### Tumor xenograft experiments and sunitinib (SU11248) treatment

Tumor xenograft experiments in nude mice were carried out as before and described in the Supplementary Information.^60^

### IHC staining and evaluation

Tumors sections from subcutaneous tumor xenografted nude mice and patients with RCC were immunohistochemically analyzed as described previously.^60^ Primary antibodies against IL-6, CD73, CD146 (Abcam, Cambridge, MA, USA), PAK1 and p-PAK1 (T423) (Santa Cruz Biotechnology) were used in the procedure.

### Statistical analyses

Experimental data were presented as mean±S.D. Student's *t-*test was used to compare continuous variables. The *χ*^2^ test or Fisher's exact test was used to compare qualitative variables. Survival was calculated from the date of surgery to the date of death or last follow-up. Kaplan–Meier method with log-rank test was applied to compare survival curves. All statistical tests were two sided and performed at a significance level of 0.05. Univariate and multivariate Cox regression models were used to analyze the impact of prognostic factors. The relationships between variables were analyzed using Spearman's correlation coefficient for correlation and *χ*^2^ test for trend. Data were analyzed using SPSS Statistics 21.0 (SPSS Inc., an IBM Company, Chicago, IL, USA) and GraphPad Prism5 (GraphPad Software, La Jolla, CA, USA).

## Figures and Tables

**Table 1:** Correlation between p-PAK1 expression and patient characteristics

**Characteristic**	**Patients (*****n*****=119)**		**p-PAK1 expression**		***P*****-value**^a^
	**Number**	**%**	**High (*****n*****=59)**	**Low (*****n*****=60)**	
*Age (years)*					0.634
≤60	51	42.9	24	27	
>60	68	57.1	35	33	
*Gender*					0.103
Male	74	62.2	41	33	
Female	45	37.8	18	27	
*ECOG PS*					0.506
0	50	42.0	23	27	
≥1	69	58.0	36	33	
*Tumor size (cm)*					**<0.001**
≤4	53	44.5	12	41	
>4	66	55.5	47	19	
*TNM stage*					**<0.001**
I	80	67.2	25	55	
II	10	8.4	8	2	
III	22	18.5	19	3	
IV	7	5.9	7	0	
*Tumor thrombus*					**0.013**
No	113	95.0	53	60	
Yes	6	5.0	6	0	
*Fuhrman grade*					**0.002**
1	42	35.3	17	24	
2	52	43.7	21	31	
3	20	16.8	15	5	
4	5	4.2	6	0	
*Necrosis*					**0.001**
Absent	55	46.2	18	37	
Present	64	53.8	41	23	

Abbreviations: p-PAK1, phospho-p21-activated kinase 1; ECOG PS, Eastern Cooperative Oncology Group performance status

*T-*test for continuous variables and *χ*^2^ test or Fisher's exact test for categorical variables. Bold values indicate *P*<0.05

a *P*<0.05 is considered statistically significant

**Table 2:** Univariate and multivariate Cox regression analysis for overall survival

**Characteristics**	**Overall survival**			
	**Univariate**		**Multivariate**	
	**HR (95% CI)**	***P*****-value**^a^	**HR (95% CI)**	***P*****-value**^a^
Age (years): ≥60 *versus* <60	1.721 (0.918–3.230)	0.091		
Gender: female *versus* male	0.788 (0.428–1.451)	0.788		
ECOG PS: ≥1 *versus* 0	1.960 (1.030–3.730)	**0.040**	1.583 (0.811–3.088)	0.178
Tumor size (cm): >4 *versus* ≤4	2.557 (1.374–4.757)	**0.003**	1.295 (0.633–2.652)	0.479
TNM stage: III+IV *versus* I+II	6.480 (3.224–13.024)	**<0.001**	3.989 (1.299–12.253)	**0.016**
Tumor thrombus: yes *versus* no	10.717 (3.867–29.706)	**<0.001**	3.042 (1.047–8.839)	**0.041**
Fuhrman grade: 3+4 *versus* 1+2	3.729 (1.931–7.202)	**<0.001**	1.018 (0.364–2.849)	0.973
Necrosis: present *versus* absent	1.563 (0.863–2.830)	0.140		
p-PAK1: high *versus* low	3.627 (1.922–6.844)	**<0.001**	2.245 (1.085–4.645)	**0.029**

Abbreviations: HR, hazard ratio; 95% CI, 95% confidence interval; ECOG-PS, Eastern Cooperative Oncology Group performance status; p-PAK1, phospho-p21-activated kinase 1

a *P*<0.05 is considered statistically significant

a Bold values indicate *P*<0.05

## Abbreviations

CDC42: cell division cycle 42
EMT: epithelial-mesenchymal transition
IFN-*α*: interferon-*α*
IL-2: interleukin-2
JNK: JUN N-terminal Kinase
MAPK: mitogen-activated protein kinase
NF-*κ*B: nuclear factor-*κ*B
PAK: p21-activated kinase
RCC: renal cell carcinoma
