# Supplementary material for: p21-activated kinase 1 determines stem-like phenotype and sunitinib resistance via NF-κB/IL-6 activation in renal cell carcinoma
Source: Cell Death Dis. 2015 Feb 12;6(2):e1637–. doi: 10.1038/cddis.2015.2 (PMC4669810; doi:10.1038/cddis.2015.2)
Supplement: Supplementary Materials and Methods [file cddis20152x1.doc]

**SUPPLEMENTARY INFORMATION**

**p21-activated kinase 1 determines stem-like phenotype and sunitinib resistance via NF-κB/IL-6 activation in renal cell carcinoma**

Yu Zhu^1,4^, Haiou Liu^2,4^, Le Xu^3,4^, Huimin An^3,4^, Weisi Liu^2^, Yidong Liu^2^, Zongming Lin^3^ and Jiejie Xu^*,2^

^1^Department of Urology, Ninth People's Hospital, School of Medicine, Shanghai Jiaotong University, Shanghai 200011, China.

^2^Key Laboratory of Glycoconjugate Research, MOH, Department of Biochemistry and Molecular Biology, School of Baskic Medical Sciences, Shanghai Medical College of Fudan University, Shanghai 200032, China.

^3^Department of Urology, Zhongshan Hospital, Fudan University, Shanghai 200032, China.

^4^These authors contributed equally to this work.

***Corresponding author.** Shanghai Medical College of Fudan University, Mailbox 103, 138 Yixueyuan Road, Shanghai 200032, China. Tel. +86 21 54237332; Fax: +86 21 64437203. E-mail address: jjxufdu@fudan.edu.cn (J. Xu)

**Supplementary Materials and Methods**

***Human samples.*** Surgical specimens were obtained from 119 patients with RCC in Zhongshan Hospital (Shanghai, China) from 1999-2004. These patients were selected randomly for this study. Histologic diagnosis revealed clear-cell carcinoma .Their histologic classiﬁcation and staging data, according to the TNM system of classiﬁcation (2010 version) was studied by two independent pathologists. Use of human tissues with informed consent was approved by the Institutional Review Board of Zhongshan Hospital.

***Transient transfection and generation of stable cell lines.*** HKC, 786-O, OS-RC-2 and ACHN cells were transfected using FuGENE HD Transfection Reagent (Roche Applied Science, Mannheim, Germany) with plasmids according to each manufacturer’s protocol. For stable transfection, transfected HKC, 786-O, OS-RC-2 and ACHN cells were maintained in DMEM or RPMI 1640 containing 800μg/mL G418 after 48 hours posttransfection. After 4 weeks of selection, individual colonies were isolated and expanded. The overexpression of PAK1 (T423E) and PAK1 (K299R) in these colonies were conﬁrmed by way of western blot analysis.

***Colony formation assay.*** Anchorage-independent growth ability was measured by using soft agar colony formation assay. HKC, 786-O, OS-RC-2 and ACHN cells per well were resuspended in DMEM containing 0.3% noble agarose (Promega Corporation, Madison, WI). This suspension was laid over DMEM containing 0.6% noble agarose in 6-well plates and further overlaid with DMEM. The plates were then incubated for 14 days in a 5% CO_2_ incubator at 37℃, with replenishment of medium every other day. Colonies were imaged using Nikon ECLIPSE TE300 and macroscopically visible colonies in 3 randomly chosen fields per well were counted for quantification.

***Cell migration assay.*** A QCM™ 24-Well Colorimetric Cell Migration Assay (Chemicon, Temecula, CA, USA) was used for this experiment. HKC, 786-O, OS-RC-2, ACHN cell lines were trypsinized and seeded at a concentration of 1×10^6^ cells/ml in serum free DMEM or RPMI 1640. Add 300μl of prepared cell suspension to each insert of a QCM™ 24-Well Colorimetric Cell Migration Assay (Chemicon, Temecula, CA, USA). 500μl medium containing 10% FBS was added to the lower chamber. The 24-Well Plates were then incubated for 24 hours in a humidified 5% enriched CO_2_ atmosphere. Cells that migrated through the 8μm pore membranes, located at the bottom of every well insert, were stained and eluted. All experiments were performed in three replicates. The number of cells migrating through the filter was counted and plotted as mean number of migrating cells per optic field (×10 magnification).

***Cell invasion assay.*** Cell invasion was evaluated using Chemicon QCM™ 24-Well Collagen-Based Cell Invasion Assay (Millipore, Billerica, MA) according to the manufacturer's instructions. HKC, 786-O, OS-RC-2, ACHN cell lines were trypsinized and seeded at a concentration of 1×10^6^ cells/ml in serum free DMEM or RPMI 1640. 300μl serum-free medium was added into the interior of each insert to rehydrate the collagen layer for 30 min at room temperature, and then it was replaced with 300 ml of prepared serum-free cell suspension containing 3×10^5^cells. 500μl medium containing 10% FBS was added to the lower chamber. Cells were incubated for 24 h at 37℃. After 24 h, all non-invaded cells were removed from the interior of the insert and the invaded cells were stained with cell stain. All experiments were performed in three replicates. The number of cells invasive through the filter was counted and plotted as mean number of invasive cells per optic field (×10 magnification). The stained cells were dissolved in Extraction Buffer and solutions were transferred to a 96-well culture plate for colorimetric reading of OD at 560 nm. The OD value represented the invasive ability.

***Flow cytometry analysis.*** For flow cytometry analysis, 786-O, OS-RC-2 and ACHN cells were fixed in 70% ethanol for 30 min at 4℃ and incubated with CD73 (FITC) and CD146 (FITC) (BD Pharmingen, San Diego, CA) for 1 h at 4°C. Analysis was performed using the BD FACSCaliburTM (Becton Dickinson). Flow cytometry data was analyzed by FlowJo software (TreeStar, Ashland, OR).

***Caspase 3/7 activity assay and Annexin V/PI staining.***  Caspase 3/7 activity assay was performed with Caspase-Glo 3/7 Assay (Promega, Madison, WI) according to the manufacturer’s instructions. Luminometer readings were taken one hour after incubation. Harvest 786-O, OS-RC-2, ACHN cells were stained with Annexin V and PI using Annexin V-FITC Apoptosis Detection Kit (BD biosciences, Rockville, MD) according to the manufacturer’s instruction. Annexin V and PI staining was determined in the BD FACSCalibur^TM^. The percentage of apoptosis cells was calculated using the Cell Fit Program.

***Tumor xenograft experiments and sunitinib (SU11248) treatment.*** Sunitinib (Biovision, Mountain View, CA) was dissolved with DMSO for 5mg/mL additive stock solution. Sunitinib gavage solution or vehicle control gavage solution was prepared freshly by combination sunitinib additive stock solution or DMSO with corn oil at a ratio of 95% corn oil: 5% DMSO. A week later, subcutaneous tumor xenografted nude mice were treated with sunitinib (40mg/kg), sunitinib (40mg/kg) in combination with IPA3（2mg/kg）, or sunitinib (40mg/kg) in combination with IL-6 neutralizing antibody (500ug/mouse) daily for 4 weeks. Tumor volumes were measured once a week after the injection with a caliper and calculated using the following formula: volume = (L×W^2^)/2, where L is length at the widest point of the tumor and W is the maximum width perpendicular to L. 6 weeks after sunitinib treatment, mice were sacrificed and subcutaneous tumor tissues were collected for analysis.

***Immunohistochemistry evaluation.*** Semi-quantitative evaluation of p-PAK1 and PAK1 expression was performed for each tumor in terms of intensity (negative, 0; weak, 1; moderate, 2; and strong, 3) and extent (0-100%). The total score of each patient was calculated by the sum of two parameters. The cutoff level of p-PAK1 was set at Median (<126, low; ≥126, high). For IL-6, CD73 and CD146 expression, the percentage of IL-6, CD73 or CD146 positive cells among the total number of cancer cells was determined. In each specimen, the IL-6, CD73 or CD146 staining were evaluated by counting an average number of 100 cells in three different fields at high magnification. A minimum of 300 cells were evaluated. The cutoff level of IL-6, CD73 or CD146 was set at 50% (<50, low; ≥50%, high). The intensity of immunohistochemistry staining in tumor cells was scored independently by two pathologists.
