# Supplementary material for: p21-activated kinase 1 determines stem-like phenotype and sunitinib resistance via NF-κB/IL-6 activation in renal cell carcinoma
Source: Cell Death Dis. 2015 Feb 12;6(2):e1637–. doi: 10.1038/cddis.2015.2 (PMC4669810; doi:10.1038/cddis.2015.2)
Supplement: Supplementary Figure Legends [file cddis20152x2.doc]

**SUPPLEMENTARY INFORMATION**

**p21-activated kinase 1 determines stem-like phenotype and sunitinib resistance via NF-κB/IL-6 activation in renal cell carcinoma**

Yu Zhu^1,4^, Haiou Liu^2,4^, Le Xu^3,4^, Huimin An^3,4^, Weisi Liu^2^, Yidong Liu^2^, Zongming Lin^3^ and Jiejie Xu^*,2^

^1^Department of Urology, Ninth People's Hospital, School of Medicine, Shanghai Jiaotong University, Shanghai 200011, China.

^2^Key Laboratory of Glycoconjugate Research, MOH, Department of Biochemistry and Molecular Biology, School of Baskic Medical Sciences, Shanghai Medical College of Fudan University, Shanghai 200032, China.

^3^Department of Urology, Zhongshan Hospital, Fudan University, Shanghai 200032, China.

^4^These authors contributed equally to this work.

***Corresponding author.** Shanghai Medical College of Fudan University, Mailbox 103, 138 Yixueyuan Road, Shanghai 200032, China. Tel. +86 21 54237332; Fax: +86 21 64437203. E-mail address: jjxufdu@fudan.edu.cn (J. Xu)

**Supplementary Figure Legends**

**Figure S1. Elevating of PAK1 kinase activity displays tumorigenic phenotype in RCC cells.** (A) Cell proliferation assay for HKC, 786-O and OS-RC-2 cells after stable PAK1 T423E transfection. Data is represented as means ± SD of triplicate experiments. **P*<0.05. (B-D) Colony formation assay (B) Cell migration assay (C) and cell invasion assay (D) for abovementioned HKC, 786-O and OS-RC-2 cells. Data is represented as means ± SD of triplicate experiments. **P*<0.05.

**Figure S2. PAK1-T423E transfection elevated the cancer stem cell markers expression in RCC cells.** Flow cytometry analysis of ALDH1, CD73 and CD146 for HKC, 786-O and OSRC-2 cells stably transfected with control or PAK1-T423E from generations G3 spheres.

**Figure S3. Inhibition of PAK1 kinase activity suppresses tumorigenicity of RCC cells.** (A) Cell proliferation assay for ACHN cells stably transfected with NS-shRNA or PAK1-shRNA stably transfected with vector (empty vector), PAK1-WT, PAK1-T423E, PAK1-K299R, and treated without or with IPA3 (7.5μM). Data is represented as means ± SD of triplicate experiments. **P*<0.05. (B-D) Colony formation assay (B) Cell migration assay (C) and cell invasion assay (D) for abovementioned ACHN cells. Data is represented as means ± SD of triplicate experiments. **P*<0.05.

**Figure S4. PAK1 kinase activity is necessary for cancer stem cell markers expression in RCC cells.** (A) Flow cytometry analysis of ALDH1, CD73 and CD146 for ACHN cells stably transfected with NS-shRNA or PAK1-shRNA stably transfected with vector (empty vector), PAK1-WT, PAK1-T423E, PAK1-K299R from generations G3 spheres. (B) Flow cytometry analysis of ALDH1, CD73 and CD146 for ACHN cells treated without or with IPA3 (7.5μM) from generations G3 spheres.

**Figure S5. PAK1 kinase activity inhibition decreases the sunitinib-induced cancer stem cell markers expression in RCC cells.** (A) Flow cytometry analysis of ALDH1, CD73 and CD146 for ACHN cells stably transfected with NS-shRNA and PAK1-shRNA-1 without or with Sunitinib (5μM), and for ACHN cells stably transfected with control (empty vector) or PAK1-K299R without or with Sunitinib (5μM) treatment, respectively, from generations G3 spheres. (C) Flow cytometry analysis of ALDH1, CD73 and CD146 for OS-RC-2 cells in the presence with IPA3 (7.5μM) with or without Sunitinib (5μM) treatment from generations G3 spheres.

**Figure S6. Sunitinib-mediated stem-like phenotype is reversed by PAK1/NF-κB/IL-6 signaling inhibition.** Flow cytometry analysis of ALDH1, CD73 and CD146 for 786-O, OS-RC-2 and ACHN cells after sunitinib with or without PDTC treatment, or with or without anti-IL-6 incubated from generations G3 spheres.
