# Supplementary figures and images for: p21-activated kinase 1 determines stem-like phenotype and sunitinib resistance via NF-κB/IL-6 activation in renal cell carcinoma
Source: Cell Death Dis. 2015 Feb 12;6(2):e1637–. doi: 10.1038/cddis.2015.2 (PMC4669810; doi:10.1038/cddis.2015.2)

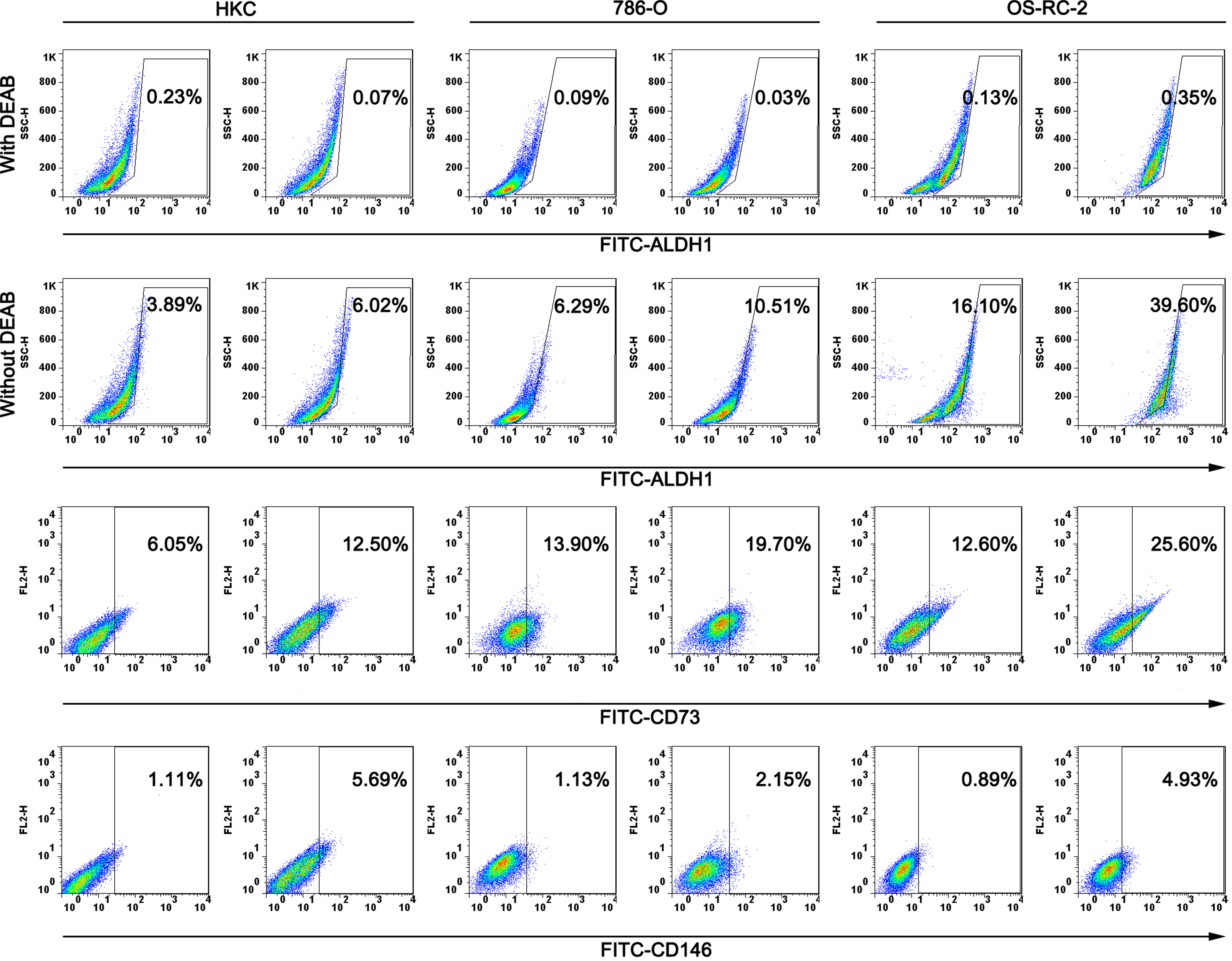

Supplement: Supplementary Figure 2 [file cddis20152x4.tif]

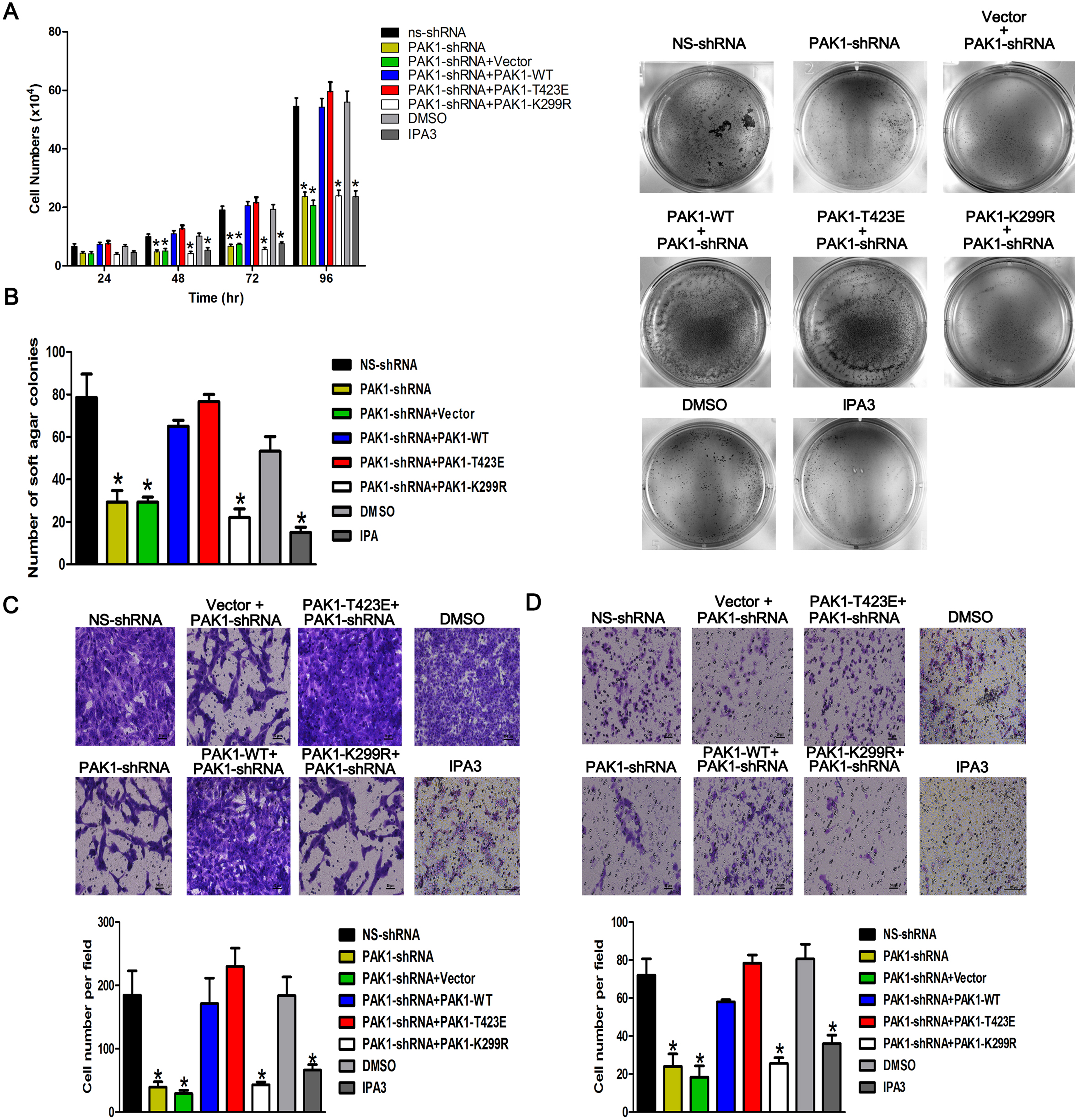

Supplement: Supplementary Figure 3 [file cddis20152x5.tif]

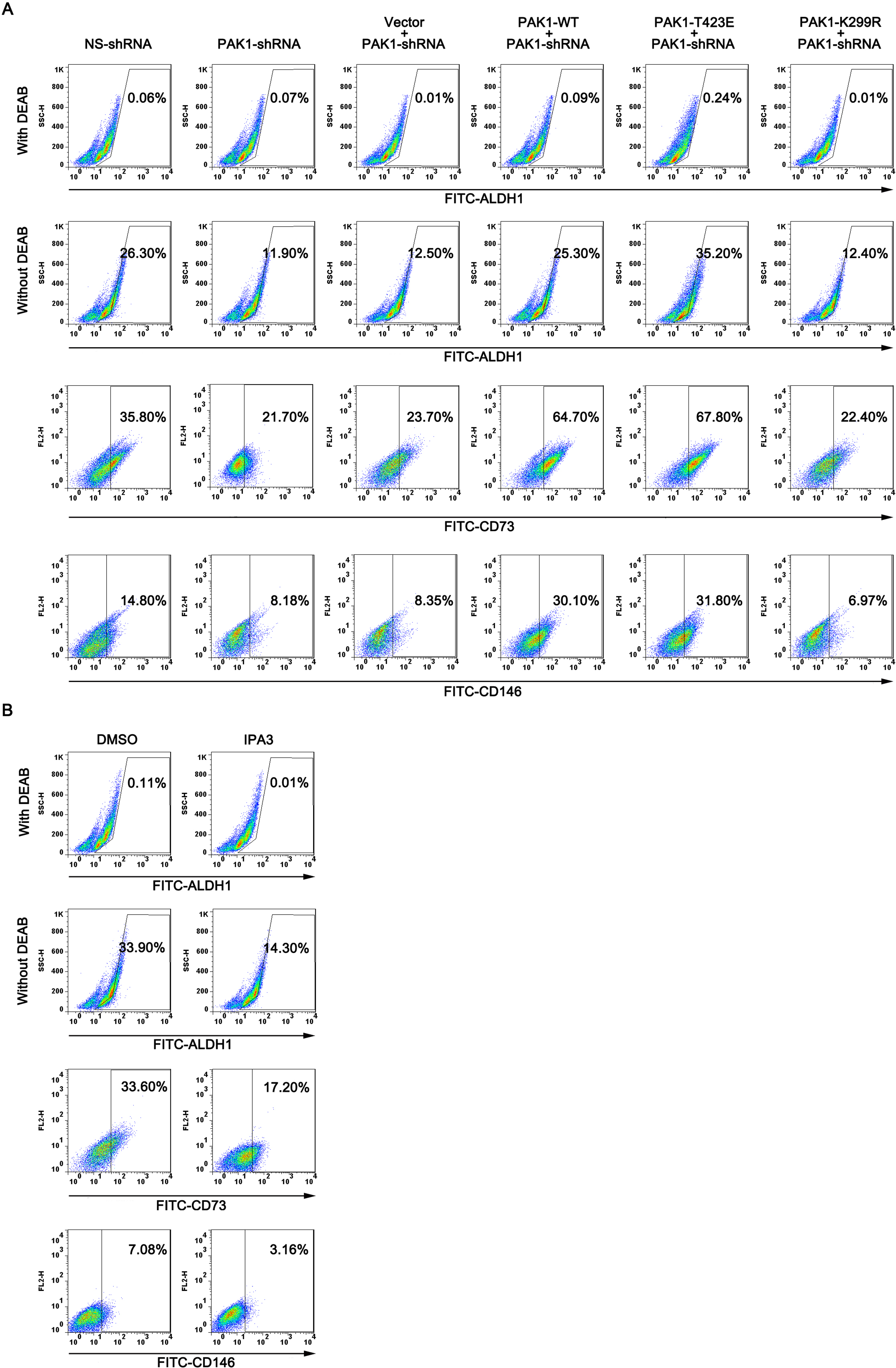

Supplement: Supplementary Figure 4 [file cddis20152x6.tif]

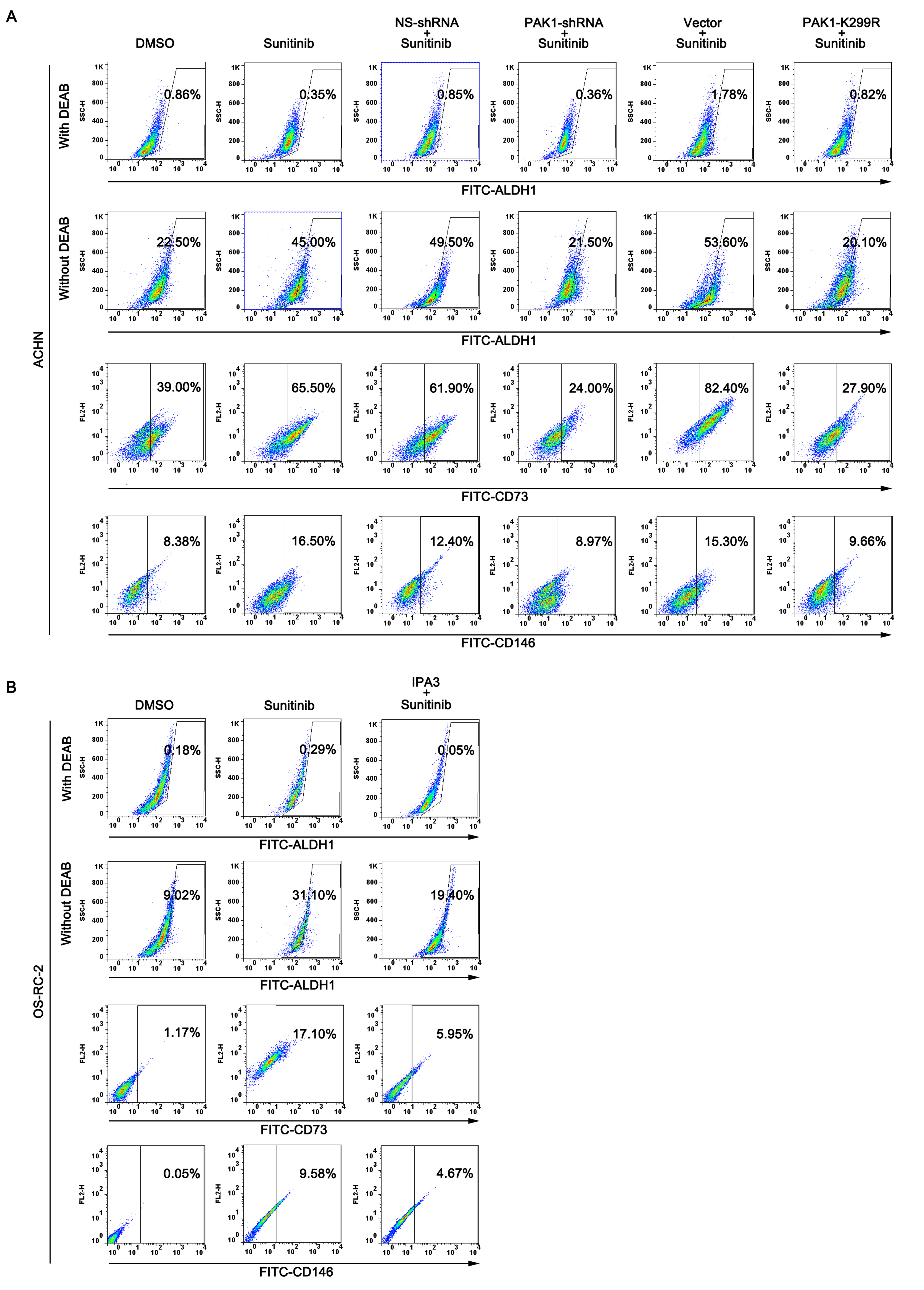

Supplement: Supplementary Figure 5 [file cddis20152x7.tif]

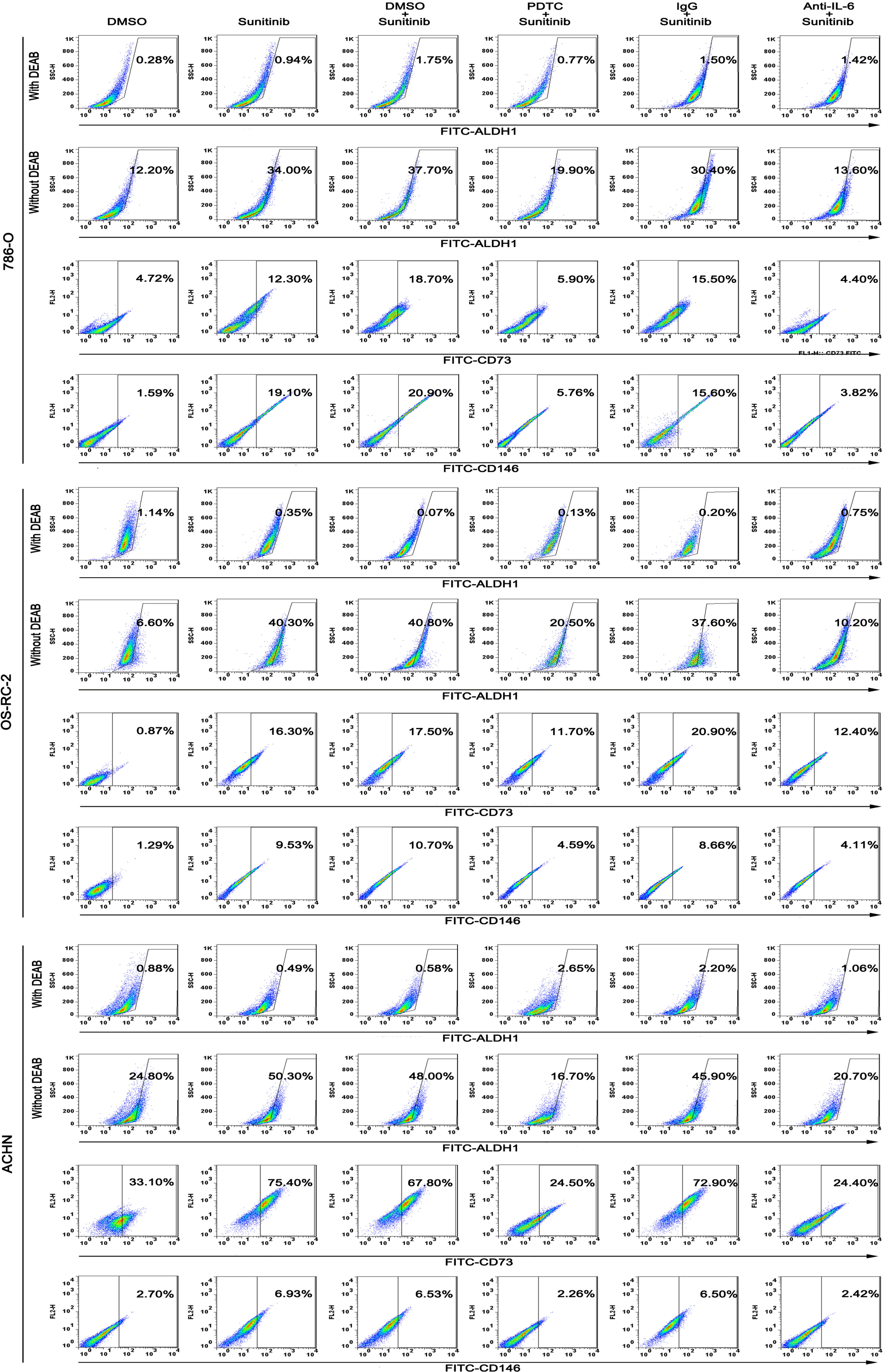

Supplement: Supplementary Figure 6 [file cddis20152x8.tif]
